# miR-140 Suppresses Tumor Growth and Metastasis of Non-Small Cell Lung Cancer by Targeting Insulin-Like Growth Factor 1 Receptor

**DOI:** 10.1371/journal.pone.0073604

**Published:** 2013-09-10

**Authors:** Yunfeng Yuan, Yaxing Shen, Liang Xue, Hong Fan

**Affiliations:** Department of Thoracic Surgery, Zhongshan Hospital, Fudan University, Shanghai, China; Texas Tech University Health Sciences Center, United States of America

## Abstract

MicroRNAs (miRNAs) are a class of small noncoding RNA molecules that play important roles in carcinogenesis and tumor progression. In this study, we investigated the roles and mechanisms of miR-140 in human non-small cell lung cancer (NSCLC). We found that miR-140 is significantly downregulated in NSCLC tissues and cell lines. Both gain-of-function and loss-of-function studies demonstrated that miR-140 suppresses NSCLC cell proliferation, migration, and invasion in vitro. Importantly, overexpression of miR-140 effectively repressed tumor growth and metastasis in nude mouse models. Integrated analysis identified IGF1R as a direct and functional target of miR-140. Knockdown of IGF1R inhibited cell proliferation and invasion resembling that of miR-140 overexpression, while overexpression of IGF1R attenuated the function of miR-140 in NSCLC cells. Together, our results highlight the significance of miR-140 and IGF1R in the development and progression of NSCLC.

## Introduction

Lung cancer is the number one cause of cancer-related death worldwide, and non-small cell lung cancer (NSCLC) accounts for at least 80% of all lung cancer cases [1,2]. Despite recent advances in the diagnosis and treatment of this cancer, the global mortality rate of NSCLC remains high, and the 5-year overall survival rate associated with NSCLC is a dismal 11% [3]. Given this, a good understanding of the molecular mechanisms underlying NSCLC development and progression is urgently needed.

MicroRNAs (miRNAs) are a class of small noncoding RNA molecules that negatively regulate the expression of target genes by either mRNA degradation or translational inhibition [4]. miRNAs can regulate the expression of a wide variety of target genes; therefore, they are involved in a wide range of biological processes including cell proliferation, apoptosis, differentiation and migration [5–7]. Recently, mounting evidence indicates that abnormal expression of miRNAs correlates with a variety of cancers, and that miRNAs can function as oncogenes and tumor suppressors [8,9]. In lung cancer, multiple miRNAs, such as let-7 family, miR-200, miR-486 and miR-146a have been identified as tumor suppressors [10–14]; on the other hand, miR-31, miR-212 and miR-196a were found to promote NSCLC carcinogenesis [15–17].

miR-140 has attracted much attention because it is involved in the development and progression of various types of cancers, including breast cancer, osteosarcoma, colon cancer and hepatocellular carcinoma [18–20]. These findings suggest that miR-140 functions as a tumor-suppressor role in these cancers; however, to our knowledge, its roles and the potential mechanisms in NSCLC remains unclear. In this study, we provide the first evidence for a role of miR-140 in NSCLC tumorigenesis and progression, and partially elucidates the molecular mechanism underlying this effect. We found that miR-140 is downregulated in NSCLC tissues and cell lines. Overexpression of miR-140 inhibited tumor growth, invasion, and metastasis of NSCLC cells. Furthermore, we identified IGF1R as a target gene of miR-140 and confirmed that miR-140 exerts its effect on the inhibition of tumor growth and metastasis by downregulating IGF1R. Our findings demonstrate a novel role of miR-140 as a tumor suppressor in NSCLC.

## Materials and Methods

### Patient samples and cell lines

Human NSCLCs and their matched normal tissues (at least 5 cm away from primary tumor) were obtained from 30 patients at Zhongshan Hospital, Fudan University (Shanghai, China). The tissues were snap-frozen in liquid nitrogen and stored at -80 °C until RNA extraction. Written informed consent was obtained from each patient and this study was approved by the Medical Ethics and Human Clinical Trial Committee at Zhongshan Hospital. Five NSCLC cell lines (A549, SK-MES-1, H157, H520 and H460) and a normal lung bronchus epithelial cell line BEAS-2B were purchased from American Type Culture Collection and cultured in DMEM (Thermo Scientific HyClone, Beijing, China) supplemented with 10% fetal bovine serum, 100 U/mL penicillin, and 100 mg/mL streptomycin (Invitrogen, Carlsbad, CA, USA). All cells were incubated in 5% CO_2_ humid atmosphere at 37 °C.

### RNA isolation and quantitative real-time PCR (qRT-PCR)

RNA was extracted from tissues and cell lines using TRIzol reagent (Invitrogen) according to the manufacturer’s instructions. The expression of mature miRNAs was assayed using TaqMan MicroRNA Assays (Applied Biosystems, Foster City, CA, USA). A two-step qRT-PCR was employed with specific primers for miR-140 designed by Applied Biosystems. U6 snRNA was amplified as an internal control. qRT-PCR analyses for *IGF1R* and *β-actin* were performed using SYBR Premix Ex Taq (Takara Bio, Dalian, China). The primers used were as follows: IGF1R forward primer, 5’- GAGAAGGAGGAGGCTGAATACCG-3’; IGF1R reverse primer, 5’-GTGATGTTGTAGGTGTCTGCGGC-3’; β-actin forward primer, 5’- AGTGTGACGTGGACATCCGCAAAG-3’; and β-actin reverse primer, 5’-ATCCACATCTGCTGGAAGGTGGAC-3’. Real-time PCR was performed using the ABI 7900 real-time PCR machine. The relative expression of each gene was calculated and normalized using the 2^−ΔΔCt^ method relative to U6 snRNA or β-actin.

### Lentivirus infection and oligonucleotide transfection

The miR-140 precursor and IGF1R siRNA were purchased from Origene (Rockville, Maryland, USA). The pre-miR-140 sequence and IGF1R siRNA sequence were cloned into pCDH-CMV-MCS-EF1-coGFP constructs (System Biosciences, California, USA). The production and purification of lentivirus were performed previously described [21]. Target cells (1×10^6^) were infected with 1×10^7^ lentivirus transducing units in the presence of 10 µg/mL polybrene (Sigma-Aldrich, St. Louis, Missouri, USA). Empty lentiviral vector was used as a control. The miR-140 inhibitor and negative control were obtained from Genepharma (Shanghai, China). Oligonucleotide transfection was performed using Lipofectamine 2000 reagent (Invitrogen) according to the manufacturer’s protocol. Cells were collected 48 h after transfection.

### Plasmid construction and luciferase reporter assays

The coding sequence of IGF1R was purchased from Origene and cloned into pcDNA3.1 (+) to generate IGF1R expression vectors. The wild-type IGF1R 3’ UTR (WT) was amplified with the following primers: 5’-ATACTCGAGTTTCCATGCAACCTCCTTCTGC-3’ (forward) and 5’-AGCAAGCTTTCCATCTTCCAAGGAGGAGGCT-3’ (reverse). Endonuclease restriction sites (*Xho*I/*Hin*dIII) were incorporated in primers to facilitate ligation into the pGL3 Basic Vector (Promega, Madison, WI, USA). Site-directed mutagenesis of the miR-140 seed sequence in the IGF1R 3’-UTR (Mut) was performed using the QuikChange™ Site-Directed Mutagenesis Kit (Stratagene, La Jolla, CA, USA). For luciferase assays, the reporter plasmid was cotransfected with a control Renilla luciferase vector into A549 and H157 cells in the presence of either miR-140 or miR-control. After 48 h, cells were harvested, and the luciferase activity was measured using the Dual-Luciferase Reporter Assay System (Promega, Madison, WI, USA).

### Cell proliferation, cell cycle, and cell apoptosis analyses

Cells (2×10^3^) were seeded in 96-well plates in 100 µl culture medium and cultured. The proliferation of the cells was assayed at the indicated time points using a CCK-8 kit (Dojindo Laboratories, Kumamoto, Japan) according to the manufacturer’s instructions. For cell cycle analysis, infected or transfected cells were fixed in 75% ethanol and stained with 50 µg/mL propidium iodide (PI). The cell cycle distribution was analyzed in a FACScan ﬂow cytometer (BD Biosciences, Bedford, MD, USA). Cell apoptosis assays were performed using an Annexin V-FITC/PI Apoptosis Detection Kit (BD Biosciences). 1×10^4^ cells were stained according to the manufacturer’s protocol and then analyzed with a flow cytometry (BD Biosciences) equipped with a CellQuest software (BD Biosciences).

### Wound healing and invasion assays

Cell migration was assessed by wound healing assays. Cells were seeded in six-well plates and cultured to 100% confluence. Wounds were generated in the cell monolayer using a plastic pipette tip. The cells were then rinsed with PBS and cultured for another 48 hours. The spread of wound closure was observed and photographed under a microscope. For invasion assays, 1×10^5^ cells in serum-free media were added into the upper chamber of an insert precoated with Matrigel (BD Bioscience). The lower chamber was filled with DMEM with 10% fetal bovine serum. After 48 hours of incubation, the cells remaining on the upper surface of the membrane was removed, whereas the cells that had invaded through the membrane were stained with 20% methanol and 0.2% crystal violet, imaged, and counted under a microscope (Olympus, Tokyo, Japan).

### Western blotting

Western blotting analysis were performed as previously described [22]. Briefly, proteins were extracted with RIPA buffer supplemented with protease inhibitors and quantified by the BCA method (Beyotime, Jiangsu, China). Lysates (25 µg) were separated on SDS-PAGE and then electrotransferred to nitrocellulose membranes (Whatman, Maidstone, UK). Membranes were blocked for 2 h at room temperature with 5% nonfat dried milk solution and then immunoblotted overnight at 4°C with primary antibodies against IGF1R and β-actin (Santa Cruz Biotechnology Inc., Santa Cruz, CA, USA). After washing, the membranes were probed with HRP-conjugated secondary antibodies. Signals were visualized with Enhanced Chemiluminescence Plus Kit (GE Healthcare).

### Animal experiments

All animal experiments were approved by the Ethical Committee on Animal Experiments of the University of Fudan Animal Care Committee, Shanghai, China. For tumor growth assays, A549 cells infected with either the miR-140-overexpressing lentivirus or the control lentivirus were injected subcutaneously into the right scapulas of nude mice (5-week-old BALB/c-nu/nu, 5 per group, 1.5×10^6^ cells for each mouse). The mice were observed over 5 weeks for tumor formation. The tumor volume (V) was monitored weekly and calculated using the formula: V = 0.5 × length × width^2^. For in vivo metastasis assays, 1.5×10^6^ A549-miR-140 or A549-miR-control cells were injected into the caudal vein of nude mice (5 per group). After 6 weeks, the mice were killed, and lung metastatic colonization was monitored and quantified.

### Statistical analysis

Data were expressed as the mean ± SD from at least three independent experiments. The difference between groups was analyzed using Student *t*-test when comparing only two groups or one-way analysis of variance when comparing more than two groups. The correlation between miR-140 and IGF1R expression was evaluated using Spearman’s correlation analysis. *P*<0.05 was considered statistically significant.

## Results

### Expression of miR-140 is decreased in NSCLC tissues and cell lines

**Figure 1:**
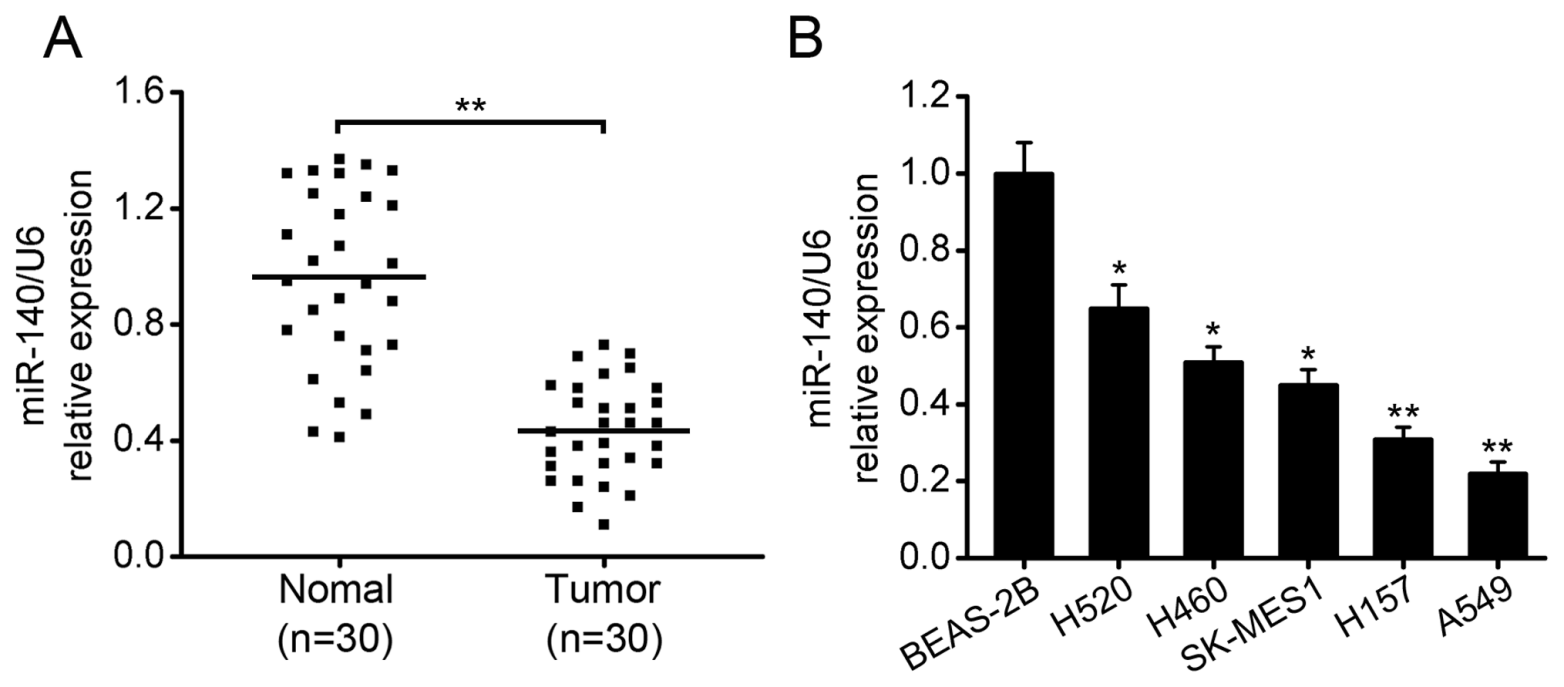
miR-140 is downregulated in NSCLC tissues and cell lines. (A) the expression levels of miR-140 in 30 pairs of NSCLC tissues and their matched normal lung tissues were measured by qRT-PCR. U6 snRNA was used as an internal control. (B) the expression levels of miR-140 in normal lung epithelial cell line (BEAS-2B) and 5 NSCLC cell lines (A549: adenocarcinoma cell line; H157, SK-MES1, H520: squamous cancer cell lines; H460: large cell cancer cell line). *P<0.05, **P<0.01.

To study the expression and significance of miR-140 in NSCLC carcinogenesis, we measured the expression of miR-140 in 30 pairs of NSCLC tissues and their matched normal lung tissues using quantitative reverse transcriptase PCR (qRT-PCR). The results showed that miR-140 expression was significantly decreased in NSCLC tissues compared with their matched normal tissues (Figure 1A). In addition, the expression of miR-140 in five NSCLC cell lines was determined. As shown in Figure 1B, the relative expression levels for miR-140 in these NSCLC cells were significantly decreased compared with that of the normal cell line BEAS-2B. Furthermore, the correlation between miR-140 expression levels and clinicopathologic parameters was analyzed. The statistical analysis revealed that the downregulation of miR-140 was significantly correlated with tumor stage and metastasis while no significant correlation was observed in other parameters (Table 1). Taken together, these results suggest that the downregulation of miR-140 may play important roles in NSCLC carcinogenesis and progression.

**Table 1:** The relationship between miR-140 expression and clinicopathologic parameters in NSCLC.

	Number	Median expression	
Characteristic	of cases	of miR-140	*P*
Age (years)			
≥60	18	0.4214±0.0468	0.4336
<60	12	0.3751±0.0291	
Sex			
Male	21	0.3972±0.0481	0.5377
Female	9	0.4226±0.0352	
Smoking status			
No	8	0.4219±0.0393	0.1834
Yes	22	0.3723±0.0436	
Histology			
AC	16	0.3854±0.0502	0.3670
SCC	14	0.3578±0.0440	
Stage			
I	7	0.5539±0.0357	0.0059
II	13	0.3737±0.0406	
III	10	0.3005±0.0251	
Metastasis			
No	13	0.4932±0.0456	0.0008
Yes	17	0.3104±0.0413	

Abbreviation: AC, adenocarcinoma; SCC, squamous cell carcinoma.

### miR-140 inhibits NSCLC cell proliferation in vitro

**Figure 2:**
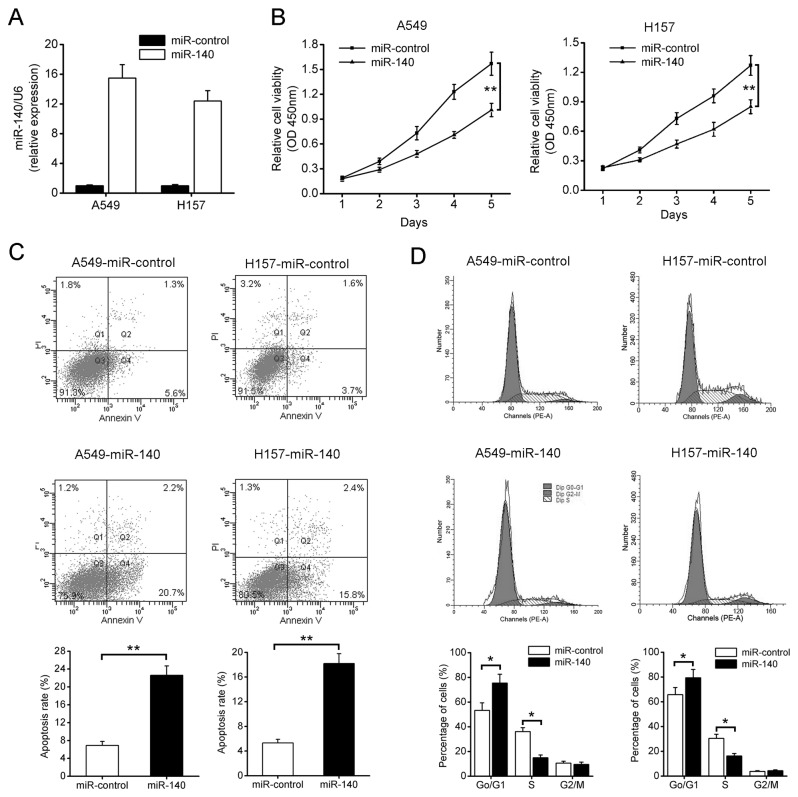
Overexpression of miR-140 inhibits cell proliferation, induces cell apoptosis and cell cycle arrest in G1 phase in both A549 and H157 cells. (A) A549 and H157 cells were infected with miR-140 or miR-control lentivirus, and the expression of miR-140 was analyzed by qRT-PCR. (B) cell viability assay (CCK-8). (C) cell apoptosis assays. (D) cell cycle analysis. *P<0.05, **P<0.01.

To better understand the role of miR-140 in the development of NSCLC, we first constructed a lentiviral vector expressing miR-140 and established stable cell lines, denoted as A549-miR-140 and H157-miR-140 after lentivirus infection. Successful overexpression of mature miR-140 in these cells was confirmed by qRT-PCR (Figure 2A). As shown in Figure 2B, overexpression of miR-140 significantly suppressed cell proliferation of A549 and H157 cells compared with their corresponding controls. miR-140 overexpression was also found to induce cell apoptosis in A549 and H157 cells (Figure 2C). In consistent with these results, miR-140 overexpression triggered an accumulation of cells at G1 phase, and decreased the number of cells at S phase in both cell lines (Figure 2D). In contrast, knockdown of miR-140 using anti-miR-140 in H520 cells promoted cell growth, while no significant change in cell cycle and cell apoptosis was detected (Figure S1). Taken together, these results demonstrate that miR-140 is able to regulate NSCLC cell growth.

### miR-140 suppresses NSCLC cell migration and invasion in vitro

**Figure 3:**
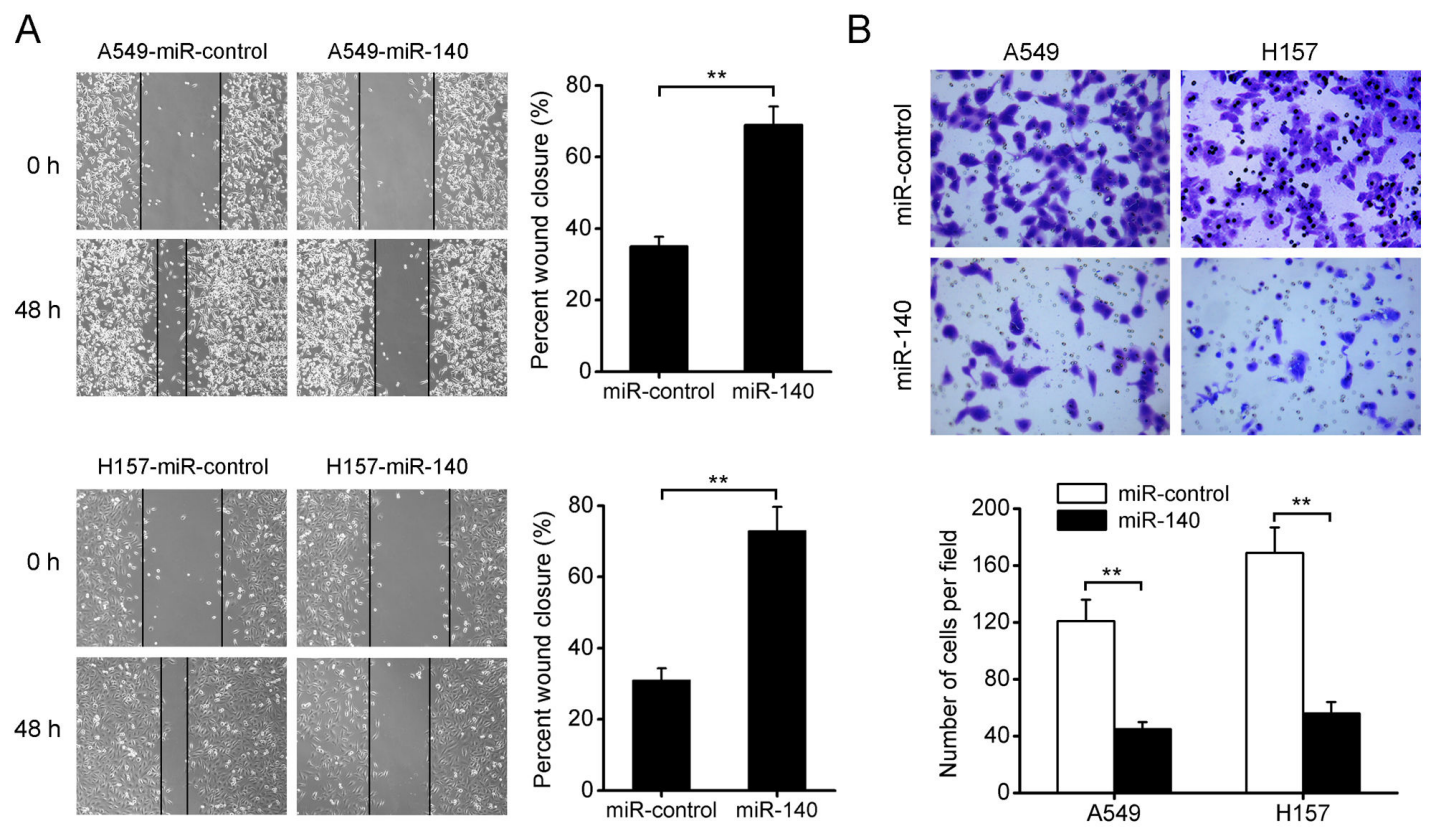
miR-140 suppresses NSCLC cell migration and invasion in vitro. The wound healing assays (A) and invasion assays (B) of A549 and H157 cells infected with miR-140 or miR-control lentivirus. The invasion assays were determined using Transwell assays with Matrigel. Magnification: 100×. **P<0.01.

We further investigated whether miR-140 could also inhibit cell migration and invasion in NSCLC. Using the wound healing assay, we found that overexpression of miR-140 dramatically suppressed tumor cell mobility in A549 and H157 cells compared with their corresponding controls (Figure 3A). Similarly, transwell assays with Matrigel demonstrated that miR-140 markedly decreased the invasive capacity of A549 and H157 cells (Figure 3B). In contrast, the wound healing and invasion of H520 cells was increased when endogenous miR-140 was silenced with anti-miR-140 (Figure S1). Taken together, these results suggest that miR-140 can suppress NSCLC cell migration and invasion in vitro.

### miR-140 inhibits tumor growth and metastasis in NSCLC cells in nude mice

**Figure 4:**
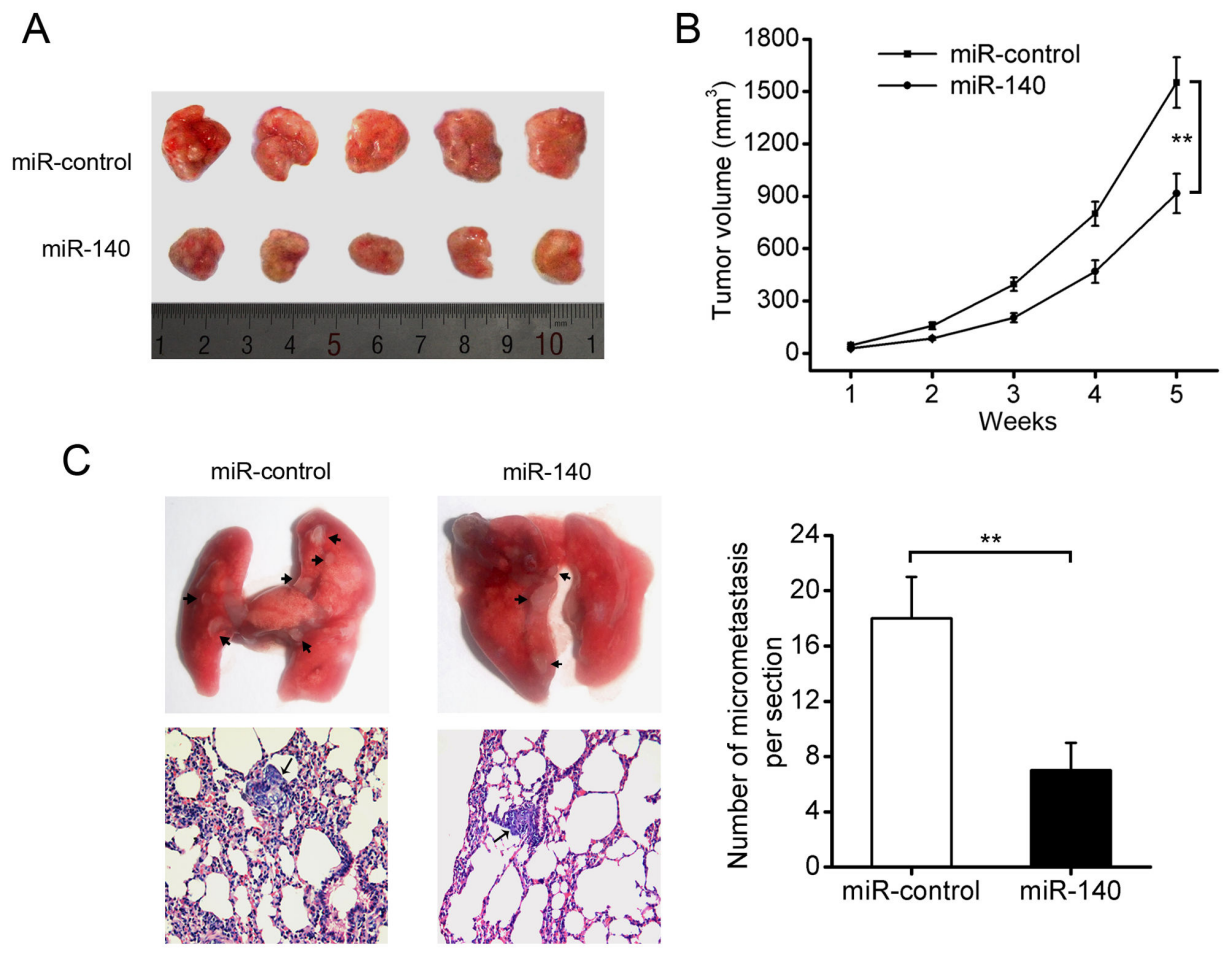
miR-140 suppresses tumor growth and metastasis of NSCLC in nude mice. (A) photography of tumors formed. (B) growth curve drawn by measuring tumor volumes on the indicated days. (C) representative H&E-stained sections of the lung tissues isolated from mice (100×). The total numbers of metastatic lesions in the lungs were counted. **P<0.01.

To further determine the effect of miR-140 on NSCLC tumor growth and metastasis in vivo, A549-miR-140 cells and A549-miR-control cells were inoculated subcutaneously into the flank of nude mice and the animals were closely monitored for tumor growth for 5 weeks. The results illustrated that miR-140-overexpressing tumors were significantly smaller in size and tumor volume compared to the control tumors (Figure 4A and B). Furthermore, we examined the effect of miR-140 overexpression on NSCLC metastasis in vivo. A549-miR-140 cells and A549-miR-control cells were injected into nude mice by caudal vein injections. The mice were sacrificed 6 weeks after injection, and their lungs were excised to observe metastatic nidi on the surface of them. As shown in Figure 4C, the number of lung metastasis nodules was dramatically decreased in A549-miR-140 group when compared with A549-miR-control group. Taken together, these results indicate that miR-140 overexpression can suppress the tumorigenesis and metastasis of NSCLC cells in vivo.

### miR-140 downregulates IGF1R by directly targeting its 3’ UTR

**Figure 5:**
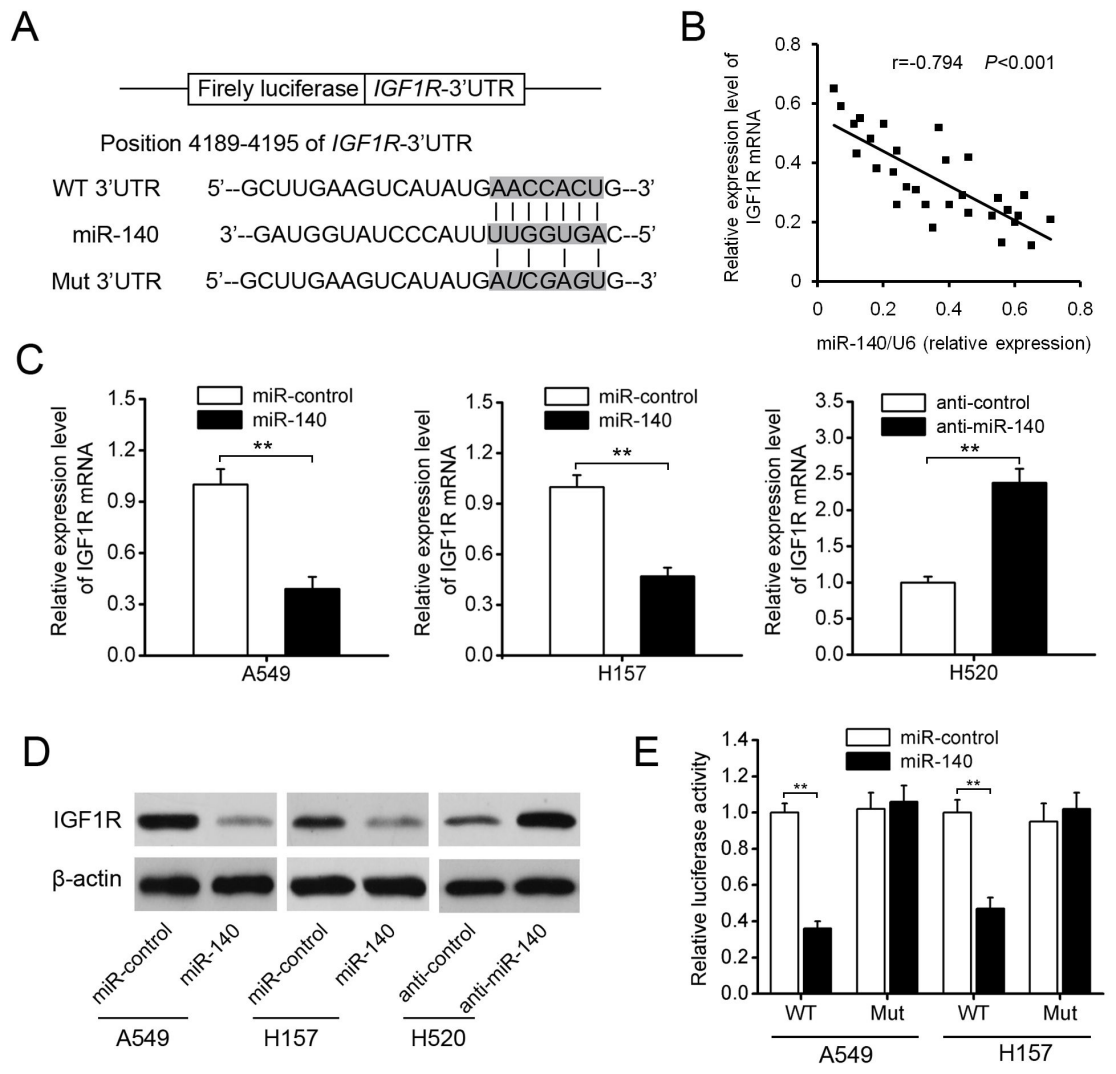
IGF1R is a downstream target of miR-140. (A) putative binding sequences of miR-140 in the IGF1R 3’ UTR. Mutation was generated in the IGF1R 3’ UTR by mutating 3 nt that is recognized by miR-140. Either wild-type (WT) or mutant (Mut) IGF1R 3’ UTR was subcloned into the dual-luciferase reporter vector. (B) a statistically inverse correlation between miR-140 and IGF1R mRNA levels in NSCLC tissues by Spearman’s correlation analysis. (C, D) the expression of IGF1R in A549, H157 and H520 cells after infection or transfection was measured by qRT-PCR and Western blotting. (E) Luciferase assay in A549 and H157 cells co-transfected with miR-140 and a luciferase reporter containing the IGF1R 3′-UTR (WT) or a mutant (Mut). Luciferase activities were measured 48 h post-transfection. **P<0.01.

To elucidate the molecular mechanisms by which miR-140 executes its function, we searched for potential targets of miR-140 using different computational methods, such as TargetScan and miRanda. In particular, we focused on oncogenes. IGF1R was identified as one candidate target of miR-140, because the complementary sequence of miR-140 was identified in its 3’ UTR by TargetScan analysis (Figure 5A). We found that the average expression level of IGF1R was significantly higher in NSCLC tissues than in matched normal tissues (Figure S2). In addition, a statistically significant inverse correlation was observed by Spearman’s correlation analysis between expression levels of miR-140 and IGF1R mRNA (Figure 5B). Furthermore, qRT-PCR and Western blotting analysis demonstrated that overexpression of miR-140 substantially decreased the expression of IGF1R in A549 and H157 cells, and that knockdown of miR-140 increased IGF1R expression in H520 cells (Figure 5C and D). To validate whether IGF1R is the direct downstream target of miR-140, a fragment of IGF1R 3’ UTR containing the putative miR-140 binding site was cloned into a luciferase reporter vector. Luciferase reporter assays showed that up-regulation of miR-140 significantly decreased the relative luciferase activity of IGF1R-3’UTR in A549 and H157 cells, but had no effect on the mutant of IGF1R-3’UTR (Figure 5E). Taken together, these results suggest that miR-140 down-regulates IGF1R expression by directly targeting its 3’ UTR.

### IGF1R is involved in miR-140-induced suppression of NSCLC cell growth and invasion

**Figure 6:**
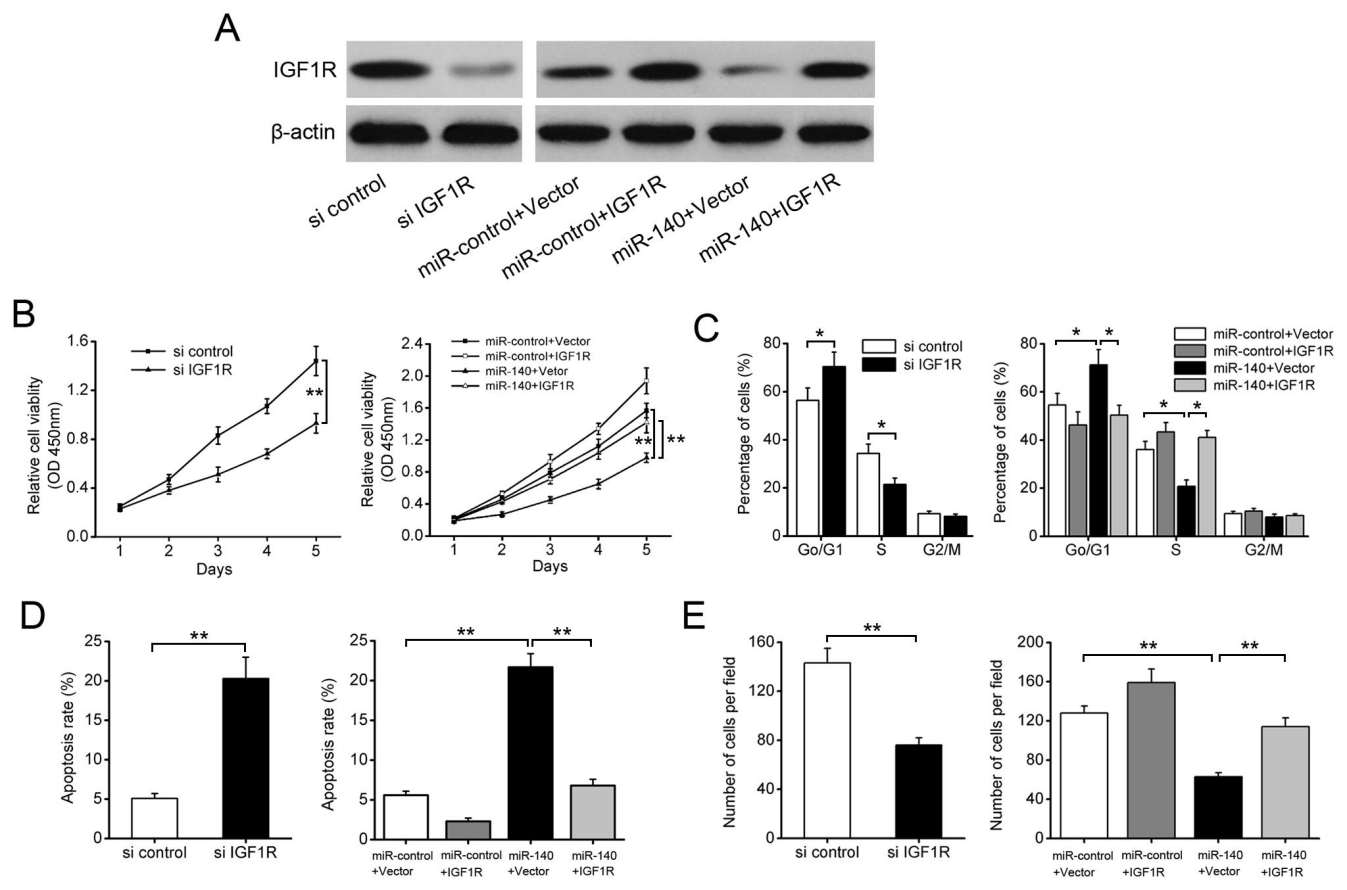
IGF1R is involved in miR-140-induced growth and invasion inhibition in A549 cells. A549 cells were infected with specific si IGF1R, or transfected with IGF1R plasmid lacking 3’ UTR along with miR-140. (A) Western blotting analysis. (B) cell viability assay (CCK-8). (C) cell cycle analysis. (D) cell apoptosis assays. (E) Transwell invasion assays. *P<0.05, **P<0.01.

To further examine whether miR-140 exerts its tumor suppressor function through downregulation of IGF1R, we performed gain-of-function and loss-of-function analyses. Firstly, A549 cells were infected with lentiviral constructs containing IGF1R siRNA or the negative control. Western blotting analysis confirmed that the expression of IGF1R was suppressed (Figure 6A). As expected, IGF1R knockdown significantly inhibited cell growth, induced G1 arrest, and increased apoptosis in A549 cells (Figure 6B, C and D). Furthermore, Transwell assays indicated that IGF1R downregulation suppressed cell invasion of A549 cells (Figure 6E). These results were similar to the effects of miR-140 overexpression. Subsequently, A549-miR-140 cells were transfected with IGF1R plasmids lacking 3’ UTR. As shown in Figure 6B, C and D, overexpression of IGF1R significantly rescued miR-140-induced cell growth inhibition, cell-cycle arrest and apoptosis. The inhibitory effect of miR-140 on cell invasion was also antagonized by IGF1R overexpression (Figure 6E). Taken together, these data demonstrate that IGF1R is a functional target of miR-140.

## Discussion

miRNAs have been reported to play important roles in carcinogenesis and tumor progression [23,24]. Alterations of miRNAs expression are implicated in almost all aspects of cancer biology, including cell growth, apoptosis, migration and/or invasion, and they can function as either tumor suppressors or oncogenes [25]. In the present study, we focused on miR-140, which has been indicated to be a possible tumor suppressor in human malignances. Song et al. showed that overexpression of miR-140 inhibited cell proliferation in both osteosarcoma and colon cancer cell lines [19]. More recently, Yang and colleagues reported that miR-140-5p is significantly decreased in HCC tissues and cell lines, and its overexpression suppresses tumor growth and metastasis by targeting transforming growth factor β receptor 1 and fibroblast growth factor 9 [20]. To date, however, the role of miR-140 in NSCLC carcinogenesis and the molecular mechanisms by which miR-140 exerts its functions remain unclear.

In this study, we showed that miR-140 expression was significantly decreased in NSCLC tissues and cell lines. Overexpression of miR-140 could effectively inhibit NSCLC cell proliferation, induce cell cycle arrest, enhance apoptosis, and suppress tumor growth in nude mice. Furthermore, miR-140 overexpression significantly repressed cell motility and invasion in vitro and tumor metastasis in vivo. Accordingly, knockdown of miR-140 promoted cell proliferation and invasion. These results suggest that miR-140 might be a novel tumor-suppressor miRNA in NSCLC.

To elucidate the underlying mechanisms involved in the miR-140-induced inhibition on NSCLC growth and metastasis, we used different prediction algorithms to predict gene targets for miR-140. The insulin-like growth factor-1 receptor (IGF1R) oncogene, which is frequently overexpressed in many malignancies and functions as an important regulator of cell proliferation, survival, and metastasis [26–29], was identified as a critical downstream target of miR-140, and this conclusion is supported by the following evidence: (A) complementary sequence of miR-140 is identified in the 3’ UTR of IGF1R mRNA; (B) overexpression of miR-140 significantly reduced IGF1R levels in NSCLC cells, whereas knockdown of miR-140 enhancedIGF1R expression; (C) miR-140 overexpression reduced the activity of a luciferase reporter containing the wild-type 3’ UTR of IGF1R mRNA; (D) the inhibitory effects of miR-140 on NSCLC cell proliferation, apoptosis, and invasion were reversed by overexpression of IGF1R; (F) IGF1R was upregulated in NSCLC tissues and inversely correlated with the expression levels of miR-140. Together, these data strongly suggest that miR-140 inhibits NSCLC growth and metastasis through downregulating IGF1R.

In conclusion, the present study showed that miR-140 is significantly downregulated in NSCLC tissues and cell lines. Overexpression of miR-140 inhibits tumor growth and metastasis of NSCLC through directly targeting IGF1R. Our data suggest that the frequently downregulated miR-140 leads to the increased expression of IGF1R and in turn contributes to the development and progression of NSCLC.

## Supporting Information

Figure S1 **Konckdown of miR-140 promotes cell proliferation, migration, and invasion of H520 cells.** (A) H520 cells were transfected with anti-miR-140 or anti-control, and the expression of miR-140 was analyzed by qRT-PCR. (B) cell viability assay (CCK-8). (C) cell apoptosis assays. (D) cell cycle analysis. (E) Wound healing assays. (F) Transwell invasion assays. *P<0.05, **P<0.01.
(TIF)

Figure S2 **IGF1R is upregulated in NSCLC tissues.** The expression of IGF1R in NSCLC tissues and matched normal lung tissues was measured by qRT–PCR. **P<0.01.
(TIF)
