# Supplementary figures and images for: miR-140 Suppresses Tumor Growth and Metastasis of Non-Small Cell Lung Cancer by Targeting Insulin-Like Growth Factor 1 Receptor
Source: PLoS One. 2013 Sep 10;8(9):e73604. doi: 10.1371/journal.pone.0073604 (PMC3769283; doi:10.1371/journal.pone.0073604)

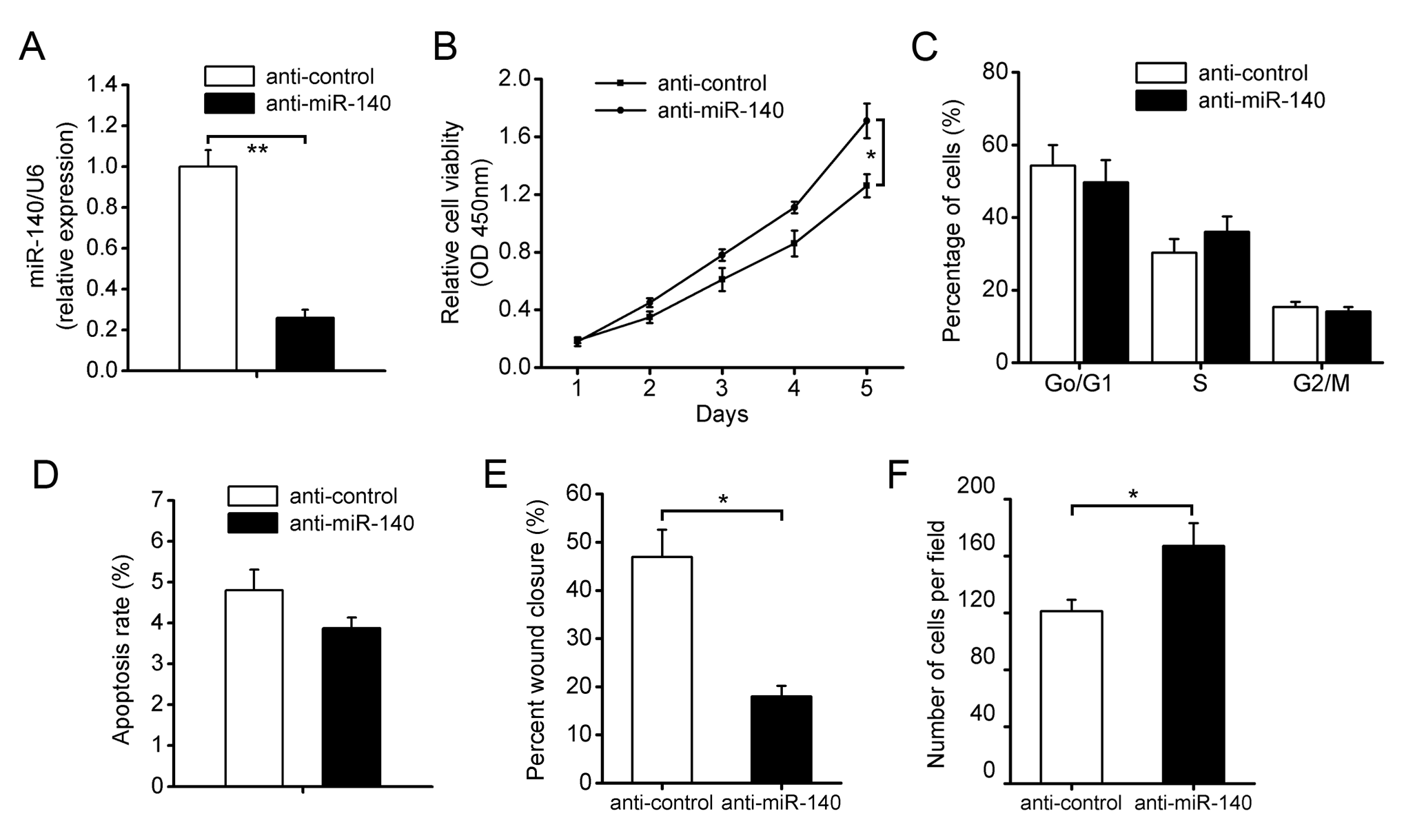

Supplement: Figure S1 — Konckdown of miR-140 promotes cell proliferation, migration, and invasion of H520 cells. (A) H520 cells were transfected with anti-miR-140 or anti-control, and the expression of miR-140 was analyzed by qRT-PCR. (B) cell viability assay (CCK-8). (C) cell apoptosis assays. (D) cell cycle analysis. (E) Wound healing assays. (F) Transwell invasion assays. *P<0.05, **P<0.01. (TIF) [file pone.0073604.s001.tif]

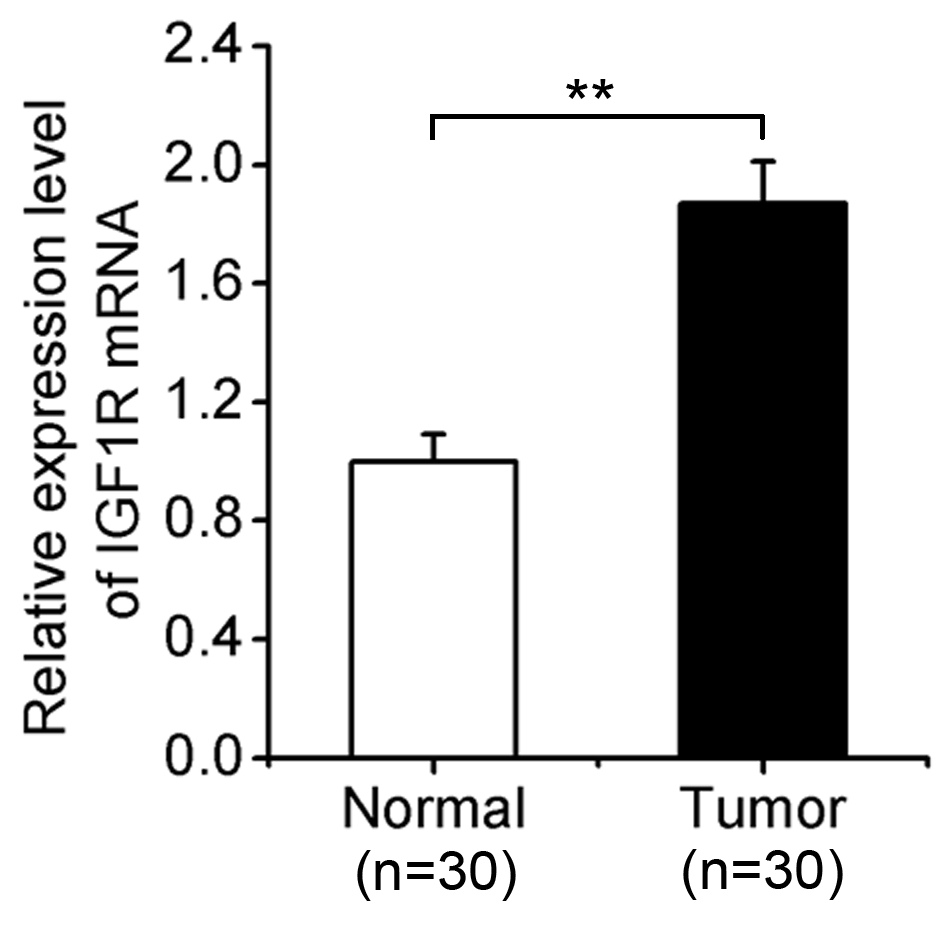

Supplement: Figure S2 — IGF1R is upregulated in NSCLC tissues. The expression of IGF1R in NSCLC tissues and matched normal lung tissues was measured by qRT–PCR. **P<0.01. (TIF) [file pone.0073604.s002.tif]
